# The Inheritance of Sex

**Published:** 1910-06-25

**Authors:** 


### The Inheritance of Sex.

The question of the origin of sex is one of the problems of the day which seems less hopeless than many others, doubtless because it can be dealt with in an experimental way. The investigations of the Cambridge School of Biologists which, under the guidance of Professor Bateson, has been studying the so-called Mendelian phenomena, have yielded results that, if not as satisfactory as was hoped by the more enthusiastic workers, are at least tangible and valuable. It is at present impossible to decide whether the sex is predetermined in the egg or whether external conditions effect a change. Modern biologists favour the view that sex is already predetermined, but this statement does not really remove the difficulties of the matter. If the sex is so predetermined, what, then, was the predetermining cause? Either it was environment or else it was an inscrutable destiny which had arranged the matter. In fact, those who maintain that variations are discontinuous and pretend to see in Weissmann's theories the last word on the subject of inheritance, must accept a blind chance as the underlying principle of heredity, whether of sex or other characteristics. To speak of the struggle in the germ between "ids" and "determinants," is a terminological prevarication which under the guise of the vicious, if verbose, circle pretends more than it can attain. Striking as are the experiments of Doncaster with the moth *Abraxas grossulariata* and the experiments of Miss Durham with canaries, from which maleness and femaleness seem to depend on the presence or absence of some other quality, it must be admitted that at present they offer no solution of the real problem. What we wish to know eventually is whether there may not be some greater external force which causes the predetermination in the egg. It has been stated, for example, in a general way that after a war in a country considerably more males are born than females in order to fill up the losses caused by disease and the enemy's weapons. There is, however, no authenticated instance on which such a generalisation can be based, though statistics should be available from Japan. A point which experimenters with animals and plants are inclined to overlook is the fact that psychological influences in man are probably far more potent, and that, even if Lamarck's dictum "that animals acquired new organs by wishing for them" be inapplicable to the animal world, it has far more significance when considered in the light of the exceptional development of man. An examination of 26 genealogies set out in Foster's well-known work on Yorkshire families did not lead to any definite conclusions, although the time ranged from 1399 to 1827. Out of a total of 1,186, 706 were male, 480 female. In most cases children that died in infancy were included, so that the total is not as erroneous as might be expected. During no period, however, did there seem to be a definite ratio between the sexes. Sometimes in one family there were all boys, sometimes twice as many girls as boys, sometimes the sexes were numerically equal. Even where a family rich in males married into a family of greater female productivity no marked change was observable in the ratios of the sexes. However vague it may appear, it is quite possible that some law, as widely applicable as that of gravitation, is discoverable beneath the complexities which beset the problem of sex-origin.

